# Trends in Trust, Safety, and Health Service Access Among Women Participating in an Antiviolence Outreach Program: Protocol for a Mixed Methods Study

**DOI:** 10.2196/88265

**Published:** 2026-04-27

**Authors:** Vicky Bungay, Phoebe M Long, Adrian Guta, Scott Comber, Lady Laforet, Patricia Tait

**Affiliations:** 1 Capacity: The Centre for Research in Community Engagement and Gender Equity, School of Nursing, University of British Columbia, Room 5280A - 5955 University Blvd, Vancouver, BC, V6T 1Z1, Canada, 1 604 822 7933; 2 School of Social Work, University of Windsor, Windsor, ON, Canada; 3 Faculty of Management, Rowe School of Business, Dalhousie University, Halifax, NS, Canada; 4 Welcome Centre Shelter for Women and Families, Windsor, ON, Canada

**Keywords:** gender-based violence, implementation science, community-based participatory research, health services, women, gender equity, convergent mixed methods

## Abstract

**Background:**

Women who are impacted by diverse forms of violence and structural disadvantage such as poverty, health inequities, and precarious housing experience significant barriers to health care. Outreach is a promising strategy to mitigate barriers to care. Until recently, outreach has focused on women’s behaviors, with less attention paid to the intersecting systemic inequities inclusive of stigma and discrimination, poverty, and compartmentalized health service delivery models that impact care engagement and access.

**Objective:**

The research study aims to (1) describe the demographic characteristics, baseline health status, and care access among diverse women impacted by gender-based violence; (2) explore preliminary changes in trust in the outreach program over time; (3) explore trends in participants’ access to health and social care services and safety planning over time; and (4) explore the contextual factors impacting trends in trust, service access, and safety.

**Methods:**

An exploratory outreach intervention will be conducted in 2 Canadian cities in partnership with community-based service organizations focused on housing security and victim support. Participants will be women eligible for these services who are experiencing barriers to timely and appropriate health and social services commensurate with their self-identified needs. The analysis will adopt a convergent mixed methods design in which quantitative and qualitative data will be collected concurrently and subsequently analyzed in parallel and then merged for data integration to fully contextualize study findings. Data will include surveys conducted at up to 4 time points to assess service access, trust and safety planning, and qualitative interviews with participants detailing sociostructural and individual factors impacting service access and trust and safety planning. Case notes will be recorded for all outreach engagement with participants. Descriptive statistics and data visualization analytic techniques will be used to document demographic characteristics and trends in trust, safety planning, and access to and engagement with care over time. Interview data will be thematically analyzed to note contextual factors associated with safety, engagement, and trust. Data integration will be carried out to examine how observed trends are influenced by contextual features and to identify nuance in variation over time.

**Results:**

The study was funded in April 2019. Intervention implementation began in the first of 2 study hubs in October 2023 and in the second hub in October 2024, and participant enrollment was open from November 2023 to June 2025. A total of 86 women were enrolled during that time; though enrollment has now closed, data collection is ongoing and is expected to continue through January 2026. Data analysis will commence in February 2026. Results are expected in late spring 2026.

**Conclusions:**

Study results will be presented at community forums within study settings and at international conferences and will be submitted for publication in relevant journals. This study is expected to generate insight into interpersonal and structural factors shaping trends observed, extending beyond behavioral investigation with new insights into how intersecting inequities can impact trust, engagement in care, and women’s safety.

## Introduction

### Background and Rationale

Gender-based violence (GBV)—defined as harmful acts, policies, and programs directed at a person or community based on their gender [1,2]—disproportionately impacts women, girls, and gender-diverse people [2]. GBV is a global health concern, contributing to severe health inequities among those impacted, including a disproportionate burden of chronic illnesses, suicidal ideation, posttraumatic stress disorder, traumatic brain injury, cardiovascular and degenerative neuromuscular diseases, depression, and addictions [3-5]. Despite these pressing health inequities, substantial barriers to appropriate and timely care persist, resulting in worsening health, ongoing violence, high rates of emergency department use, and premature and preventable death [6,7]. Structural features of the organization and delivery of care, including inadequate and/or inappropriate services; siloed care delivery models that overlook the complexity of women’s interrelated health and social concerns (eg, traumatic brain injury, poverty, or precarious housing); and previous negative clinical encounters that exacerbate mistrust in care providers (eg, discrimination, incomplete care) are well substantiated barriers [6,8-11]. Additionally, the structural disadvantages of poverty, precarious housing, and the multiple and competing demands women juggle to address basic health and safety needs for themselves and their families (eg, securing food, shelter, and income generation) intersect with other facets of health service delivery to exacerbate barriers to care [12-15].

Outreach, as a service delivery model, is an important strategy to help improve women’s engagement with the health care system [16-20]. While a universal consensus on outreach is lacking, it is generally described as a relational practice to locate and build connections with people chronically underserved in health care with the aim of linking them to essential health services (V Bungay et al, unpublished data, 2025) [16]. Outreach tends to occur in community settings where people spend their time, such as parks, drop-in centers, on the street [16,21], in their homes, and in emergency shelters [22-25]. Within health services programs, outreach has been used effectively to foster health care engagement among people experiencing homelessness, chronic mental health issues, and/or substance use-related challenges. Harm reduction supply distribution, health promotion education, crisis intervention, infectious disease testing, and linkages to emergency shelter and health care are common outreach services [16,19,20,26], with notable benefits of sustaining engagement with mainstream health services, improving access to housing, and reducing drug-related harms [26,27]. Outreach has also increasingly been undertaken with women experiencing intimate partner violence (IPV), which is markedly reported as the most predominant form of GBV [2]. IPV-related outreach services frequently include advocacy, emotional support, linkages to health and social services, and safety planning [25,28], and evidence indicates positive outcomes of reduced violence and improved physical and psychological functioning [24,28,29].

While extant evidence indicates promising results for outreach as a health promotion strategy, much research tends to be highly compartmentalized, focusing on women’s behaviors (eg, substance use), “choices,” and motivations as barriers to care, thereby overlooking the aforementioned structural and marginalizing conditions shaping women’s experiences of GBV, health, and care engagement [9,16]. Attention to structural features of society that create and sustain conditions of GBV and poor health is critical, particularly given the documented interrelationships between systemic inequities such as racism, ableism, sexism, and classism and women’s health and health care. Indigenous women, newcomers, and women living in poverty and/or with a disability, for instance, are substantially more likely than other women to experience GBV and barriers to appropriate and timely health care [30-34]. Such health service barriers are often a direct result of stigma and discrimination within clinical encounters, which increases the likelihood of insufficient or inappropriate care, women’s reluctance to engage with care, and leaving without care being completed [34-36].

In response to some of the gaps in outreach as a health service intervention specific to women experiencing GBV, we have witnessed increasing awareness of the relevance of trauma-informed—and trauma- and violence-informed—approaches to care engagement (Table 1). Such approaches recognize the need to create working relationships with women that build from women’s strengths and resilience and the obligations of service providers to foster safe, nonretraumatizing environments in which care occurs [28]. To date, many of these interventions have remained focused on IPV and women’s health care needs after leaving an abusive partner. While these studies are important, further intervention research is needed to advance outreach with diverse groups of women experiencing multiple and intersecting forms of GBV, particularly those experiencing the greatest violence severity, duration, and type. Moreover, as most research has focused on behavioral outcomes decontextualized from the marginalizing conditions of women’s lives, research designs that integrate data concerning structural facets of women’s experiences and their engagement with programming are urgently needed. Therefore, we are undertaking a multisite exploratory mixed methods study of implementing a strengths-based, trauma- and violence-informed outreach model with diverse groups of women experiencing GBV. The study participants comprise women experiencing varying forms of GBV and structural disadvantage who are simultaneously not well connected to health care or social services that can address their priority needs. We hypothesize that by centering women’s strengths and rights to self-determination and offering outreach through a harm reduction and trauma- and violence-informed care way, women will build working relationships with outreach workers over time, and we will explore how these relationships may influence changes in trust with outreach teams, women’s care engagement, and capacity for safety planning over time. These study findings may contribute to further development of outreach models that avoid siloed approaches to service delivery while supporting women’s rights to self-determination.

**Table 1.** Guiding principles for community-led outreach and engagement (CLOE).

Guiding principle	Definition	Implications for outreach practice
Tackle gender-based violence	GBV^a^ is the harmful acts, practices, policies, and social stratifications that are directed at an individual or group based on their gender [15,37-39]. GBV is both structural and interpersonal. Sociostructural facets of society contribute to and create conditions of interpersonal violence inclusive of physical, psychological, and material acts of violence that are disproportionately experienced by women as a group, and particularly among those impacted by systemic inequities operating through racist, ableist, and class-based policy and practices [37].	Outreach workers understand that violence against women is a global issue perpetuated through social norms that impact how services are organized. Outreach workers work to reduce the negative effects of violence in women’s lives, inclusive of stigma and discrimination. Safety planning in everyday life activities and for health care encounters is an important element of outreach practice.
Foster relational engagement that respects a rights-based approach to personhood	Personhood encompasses people’s strengths, capacities, and inherent rights and capabilities to make their own choices. A relational approach to personhood challenges limiting, deficit-oriented views of women that can perpetuate ideologies of deviance or foster paternalism in care.	Outreach is client-centered and women-led. Outreach workers and women work collaboratively to set timing and frequency of engagements, priority health concerns, and strategies to address these concerns.
Practice a relational approach to harm reduction	Harm reduction is a relational practice that emphasizes respect and nonjudgmental approaches toward women’s use of substances while addressing the broader sociostructural context (eg, poverty, violence, discrimination, criminalization) shaping their substance use and health opportunities [40].	Outreach workers understand the interrelationship between GBV, trauma, and substance use. Outreach workers have a basic understanding of the impact of substance withdrawal for women’s overall health and well-being. Technical tools such as harm reduction supplies serve as mechanisms of engagement to foster reducing drug-related harm.
Practice trauma- and violence-informed engagement	TVIE^b^ has the following 6 core elements: It is vital that we understand that trauma has numerous impacts on women’s psychological and physical functioning; Women have the right to determine what constitutes safety and violence; Strengths-based approaches are critical to foster autonomy; Engagement with support workers should not cause harm; A relational approach to engagement requires collaboration and continuous negotiation of expectations within the working relationship; Trauma is situated within intersectional causes [41-43]	Outreach workers engage in nontraumatizing ways that do not require disclosure of violence. Outreach workers practice humility working with survivors, recognizing that people’s ability to fully engage fluctuates due to health and other issues. Outreach is low barrier.
Promote service integration	Service integration as a theory and model of programming and practice includes efficient, coordinated, and proactive delivery of health and human services that are tailored to the needs of individuals and communities where the services are delivered [44-49]. Embedded within systems theory, service integration requires collaboration at all levels of the “system” that impact health including factors associated with the social determinants of health. Service integration fosters appropriate and consistent service delivery along the continuum of care.	Outreach workers work at the nexus of the continuum of care and thus require knowledge about existing community services. Outreach workers are able to build effective working relationships to foster linkages across services and sectors.

a GBV: gender-based violence.

b TVIE: trauma- and violence-informed engagement.

### Study Objectives

Guided by central principles associated with rights to self-determination, trauma- and violence-informed engagement, and harm reduction, and our aims to address the impacts of GBV for women’s engagement with health care, this study’s objectives are as follows:

1. To describe the demographic characteristics, baseline health status, and care access among diverse women impacted by GBV prior to implementation of the intervention (quantitative)
2. To explore preliminary changes in trust in the outreach program over time (quantitative)
3. To explore trends in participants’ access to health and social care services over time (quantitative and qualitative)
4. To explore trends in participants’ safety planning over time (quantitative and qualitative)
5. To explore the contextual factors impacting trends in trust, service access, and safety over time (qualitative)

### Theoretical Approaches and Evidence Base

The current study builds from an existing program of research aimed at improving health service delivery for women impacted by GBV and structural disadvantage, inclusive of stigma and discrimination in health care encounters [14,16,50,51]. Critical perspectives concerning marginalization, equity, inclusion, and social justice inform all aspects of this work. For our purposes, marginalization refers to the structural disadvantage associated with intersecting inequities, such as poverty, racism, ableism, sexism, stigma, and discrimination [1,14]. This structural disadvantage operates as socially and historically situated exclusion of individuals and communities from political, material, and economic resources that influence their opportunities for optimal health. Marginalization is often implicitly sanctioned, justified by beliefs and perspectives that create and sustain privilege of some in ways that subordinate and discriminate against those who are marginalized. Consequently, marginalization is deeply entwined with GBV and its negative effects for women [1,8,14,52]. For example, social norms that create and sustain the “feminine” as subordinate to “masculine” contribute to victim blaming of female survivors, which negatively impacts women’s likelihood to seek health care and/or other forms of support when victimized by violence [1]. Moreover, gendered pay-equity gaps sustain women’s poverty, thereby reducing options and opportunities for health. Such understandings of marginalization do not negate the agency of individuals. Given the socially constructed facets of marginalization, however, agency is understood as a series of social processes embedded in historical and social contexts in which people’s opportunities are activated in different ways depending on circumstances and context [37]. In keeping with our goals to tackle structural disadvantage and the related barriers to care (eg, stigma and discrimination in care encounters; siloed service delivery models) and our commitment to inclusive and equity-oriented approaches to service delivery, we codeveloped a holistic outreach model known as the community-led outreach and engagement (CLOE) intervention detailed below.

### The CLOE Intervention

Health interventions, particularly those that aim to ameliorate health inequities among groups experiencing marginalization, are noticeably more effective when designed and implemented through fulsome community engagement [53]. Thus, our study draws on central tenets of community-based and participatory research approaches, including tackling core issues contextualized to the local communities in which the research is situated, integrating inclusive research teams comprised of community service leaders and staff, women with lived experience of GBV and academic researchers, and commitments to equity and justice. Together, these teams have undertaken a series of studies to inform the study protocol. Earlier exploratory research and systematic reviews identified 5 core principles underpinning outreach practice that were necessary to build and sustain working relationships between outreach service providers and women experiencing GBV [14]. These principles are detailed in Table 1. Research has also identified the core strategies to translate these principles into practice, the net results of which enabled women’s trust in the outreach teams as competent, respectful, and safe clinicians who could facilitate women’s engagement with essential health and social care services. These studies show promising exploratory results for improved health care engagement, including increased ease of accessing health care, obtaining a consistent primary care provider, enhanced sense of self-worth, and increased capacity of women to more autonomously navigate health and social care services (V Bungay et al, unpublished data, 2025) [54].

## Methods

### Overview

This exploratory study includes a 1-year outreach intervention across 2 study settings with women with lived or living experience of GBV. Given the exploratory nature of the study concerned with trends and context, a convergent mixed methods design [55] will be used to address the 5 objectives outlined above. Convergent designs enable researchers to collect and analyze quantitative and qualitative data separately and in parallel, merging and comparing results for a comprehensive understanding of trends observed during the intervention period and contextual features of participants’ experiences influencing trends observed [56]. Surveys and interviews with women who enroll in the project and participant case notes concerning their engagement with outreach workers comprise the data sources collected throughout the intervention period, which is expected to last from November 2023 to January 2026. Specifically, for our exploratory intervention study, surveys will provide the quantitative breadth by systematically gathering structured data with the entire sample, while the interviews will offer qualitative depth by allowing participants to articulate their experiences in their own words and through their own interpretations. This protocol was developed in accordance with American Psychological Association Style Journal Article Reporting Standards: Mixed Methods Article Reporting Standards.

### Client/Patient and Public Involvement

The design and implementation of the study adhere to core principles of community-based participatory research [14,57,58]. As such, the research team represents a coleadership model inclusive of community service managers and staff, women with lived and living experience of GBV, and researchers with quantitative and qualitative expertise in women’s health and health care. Specifically, the need for a novel women-led, strengths-based, and trauma- and violence-informed outreach intervention was identified by community service leaders, staff, and women with lived or living experience [14]. A community advisory committee comprised of women with lived or living experience provided guidance and expertise on outreach strategies and training for interventionists. Community service managers contributed to protocol development, tailoring the intervention to their local context, and integration of the study into their existing programming. Named research partners will support delivery of the intervention, data collection and analysis, and be active in the dissemination of the results to appropriate stakeholders inclusive of peer-reviewed publications.

### Settings

The study intervention settings, hereafter referred to as study hubs, include 2 nonprofit organizations in 2 Canadian provinces, detailed in Table 2. Each organization serves diverse groups of women with lived or living experience of GBV through central feminist principles of gender equity. The study hubs were purposely selected to maximize existing partnerships within the research team and the diversity of settings with established credibility for serving women impacted by GBV in their communities. Intervention activities have been tailored to the local contexts of the 2 community-service organizations, which are focused on providing various supports ranging from emergency and short-term shelter, outreach and education, harm reduction programming, and legal and victim-specific services. Intervention tailoring also considers the unique services and contexts of 2 different urban centers: 1 mid-sized city in East-central Canada, with an economy largely driven by manufacturing and its strategic location as a major US-Canada border crossing and hub for the auto industry; and 1 smaller city and outlying rural area in Western Canada, with economic ties to agriculture, tourism, and manufacturing.

**Table 2.** Overview of study hubs: key contextual factors.

Study hub	Population served	Service focus
*Hub A: Central Okanagan Elizabeth Fry Society,* Kelowna, British Columbia	Survivors of intimate partner violence, sexual assault, and child abuse	Situated on the unceded territory of the Syilx Okanagan people Serves people throughout the Central Okanagan Valley Provides a variety of services, including specialized victim assistance, crisis response and risk assessment, safety planning, court support, sexual assault counseling, Indigenous victim services, community education, and peer support groups
*Hub B: Welcome Centre Shelter for Women & Families, Windsor, Ontario*	Self-identifying women+^a^, families, and children experiencing homelessness and related challenges	Located on the traditional territory of the Three Fires Confederacy of First Nations, which includes the Ojibwa, the Odawa, and the Potawatomi Provides programs and services to those experiencing homelessness in Windsor-Essex County Operates under Housing First as a core philosophy, focusing on evidence-based practices to address chronic homelessness, with low-barrier and trauma-informed programming incorporated throughout Provides emergency shelter, housing support, family support, harm reduction services, peer engagement, medical care, and food services

a Women+ refers to the Centre’s inclusive understanding of women, including cis-gender women, transgender women, and nonbinary individuals.

### Intervention

#### Overview

The 1-year CLOE intervention will be delivered by intervention outreach workers (IOWs) with expertise in providing health and social care navigation support to women affected by GBV. Prior to implementation, IOWs will undergo online and in-person training developed and delivered by members of the research team on the following topics: outreach practice that adheres to the core principles underpinning the intervention, emergency mental health first aid, crisis intervention, professional boundaries, safety planning, and strategies for self-care and wellness. Additional training specific to the research site models of care (eg, IPV and sexual assault victim services training; housing policies; Naloxone administration) will be provided by the community service organizations.

Once a participant enrolls (see more details in the Recruitment Process section), they will be assigned to an IOW by the project coordinator. The participant and assigned IOW will work collaboratively to complete a standardized assessment of core health, safety, and well-being domains in keeping with the core domains of support provided by the outreach team, which are detailed in Table 3. These assessments will enable participants and outreach workers to identify women’s current strengths and barriers in navigating health care and social services, their short-, medium-, and long-term goals to foster health and safety, and the support needed to attain these goals. Unlike intake processes that regularly occur in a static clinical setting, the assessments will be women-led and therefore completed at a location and pace that works for the participant [14]. Goal setting, planning, and activity implementation (eg, engaging with necessary health and social care services) will be incremental starting with short-term, easily attained goals (eg, obtaining lost identification; applying for shelter) to foster relationship building between IOWs and participants and to promote confidence and enhance capacity for addressing more complex goals [59]. As the relationship strengthens and trust and capacity increase, participants and IOWs will shift toward medium- (eg, retaining housing, obtaining a primary care clinician) and longer-term (eg, substance use health care, vocational training) goals. The mode of communication (eg, in-person, text, email, phone), timing, and location of engagements with IOWs will be codetermined based on participant comfort, access to technology, and preferences. No minimum number of engagements will be required to sustain enrollment. Participants who miss a connection—defined as not meeting an IOW at a predetermined time and place—will receive follow-up by IOWs to assess safety and well-being and to reschedule any planned activity. Participants and IOWs will undertake quarterly (every 3 mo) and ad hoc reviews of activities, goals achieved, next steps, and reassessment for new and emerging priorities contextualized to life events (eg, loss of housing; recent assault). Safety planning—defined as engaging with participants to explore threats to personal, psychosocial, physical, and material safety that can be lessened through specific actions within an individual’s control—will be integrated into all aspects of assessment, planning, and health and social care navigation activities. As violence is both structural and interpersonal, safety planning will focus on acts of violence in the form of stigma and discrimination in the context of health and social care encounters and when moving about in public spaces and interpersonal acts of violence by intimate and, where appropriate, commercial sex partners. We will also attend to safety in the context of substance use to promote women’s safety when using substances alone or with others.

**Table 3.** Outreach domains of practice and support.

Outreach support domain	Definition
Financial security	Financial services necessary for everyday life such as banking, income assistance, income tax, financial grants, peer work, and employment or other income sources
Education or vocational support	Identifying, accessing, and receiving resources and support for continuing education or employment counseling, training, or hiring
Food security	Gaining or ensuring access to food, including accessing food banks and meal programs
Housing security	Housing services necessary to address precarious housing, homelessness, safety at home, and adequacy of current housing
Transportation security	Getting or securing immediate or ongoing access to transportation, and enhancing confidence and independence in using transit or transportation options. This does not include transportation provided by the CLOE^a^ team.
Legal supports	Identifying, accessing, and receiving legal assistance for any legal issue (eg, parental time, family violence, parole and probation, and identification)
Family or social network	Engaging, re-engaging, or preventing contact with family or social networks, including children, other family members, friends, and acquaintances. This may include working with family services
Substance use health	Identifying, accessing, and receiving detox and treatment services; harm reduction services; and community addictions management programs (eg, methadone maintenance therapy, safe supply, etc)
Mental and physical health	Identifying, accessing, and receiving home, acute, emergency, and primary care health services specific to new and chronic physical and mental health problems
Safety planning and support	Identifying threats to personal physical, psychological, and material safety and developing plans to mitigate these threats through specific actions within an individual’s control

a CLOE: community-led outreach and engagement.

#### Participants

The study population consists of self-identifying women aged 18 years or older with lived or living experience of GBV, who live or spend considerable time in the communities where the research is situated. Specific *inclusion criteria* are as follows:

- Eligible for services provided by the research site service organizations in the respective research partner communities (Hub A: survivors of IPV and/or sexual assault in their lifetime; Hub B: currently experiencing homelessness or precarious housing)
- Experiencing challenges accessing appropriate and timely health and social care services
- Self-identify as needing support to navigate health and social care that is not currently provided by the everyday services within the research sites
- Able to communicate verbally in English

#### Recruitment Process

We will use a multipronged approach to recruitment, contextualized to the service delivery models for each partnering research hub. Pamphlets and posters detailing the study purpose and the study coordinator’s contact information will be posted in general areas for both hubs (eg, waiting areas of Hub A, drop-in meal program Hub B). Outreach workers will also be available at set times in these spaces to discuss the study with potential participants and provide unconditional episodic support such as harm reduction supplies, emergency shelter referral, and meals or groceries. The study coordinator will attend staff meetings at the respective hubs and other organizations within their communities that serve the target study populations. During these meetings, they will discuss the study and provide pamphlets that staff may distribute to potential participants. Potential participants will contact the study coordinator who will also be available at set times during the week, including a range of days and evenings appropriate to potential participants’ schedules. Interested participants will contact the coordinator in person, by phone, text, or email to discuss eligibility. Eligible participants will be invited to consent to participate in the study intervention and to documentation of the activities they engage in. Consent to participate in surveys and interviews will be separate from consent to participate in the outreach intervention. That is, participants could opt to participate in the intervention, but not in other data collection activities. Consent to participate in surveys and interviews will be reaffirmed at the time of conducting the activity. If a person is not eligible, the coordinator will provide a referral to other programs provided at the hubs and/or other organizations within the community. Recruitment will be ongoing with the aim of 50 participants per hub. The sample of 50 per hub allows for in-depth exploration of participants’ experiences necessary to achieve the exploratory study aims.

### Data Collection

#### Data Sources

To assess trends in participants’ care engagement, safety planning, and overall trust with the outreach team, quantitative and qualitative data will be collected concurrently. At baseline, participants will complete a standardized quantitative assessment of their health status and health and social care service access, safety at home and in community, and their strengths in navigating health and social care (time point 0; T0); identify goals to promote health, safety, and well-being; and complete a brief survey, including health status, variables impacting health care access, and demographic characteristics. Surveys will occur at 4-month intervals (T1, T2, T3) postenrollment, inclusive of T0 variables and trust and satisfaction with the outreach team. Text-based (ie, qualitative) case notes of all interactions with the IOWs will be recorded as well as quarterly progress assessments. Qualitative interviews will occur at time point 3, focused on participants’ perspectives of strengths and challenges in participating in the intervention, intervention effectiveness in supporting their self-identified health goals, and contextual features of their everyday experiences that positively or negatively affected their engagement with the intervention, their safety, and overall access to health care. Upon study completion, participants will be transitioned to programs within research sites depending on needs and their preferences. Data sources specific to research objectives are detailed in Table 4 and an overview of data sequencing and sourcing is described in Table 5.

**Table 4.** Research questions and data sources.

Question	Data sources	Analysis
What are the demographic characteristics, health status, and care access among women impacted by GBV^a^ prior to implementation of the CLOE^b^ intervention?	Quantitative survey	Descriptive analysis
What is the impact of CLOE for participants’ trust in the outreach program?	Quantitative survey Qualitative interviews	Parallel and integrated mixed analysis
What are the trends in participants’ access to health and social services over time?	Quantitative survey Qualitative interviews Quantitative and qualitative case notes	Parallel and integrated mixed analysis
What are the trends in participants’ safety planning over time?	Quantitative survey Quantitative and qualitative case notes Qualitative interviews	Parallel and integrated mixed analysis
What are the contextual factors that influence the trends in trust, safety, and care access observed over time?	Quantitative and qualitative case notes Qualitative interviews	Parallel and integrated mixed analysis

a GBV: gender-based violence.

b CLOE: community-led outreach and engagement.

**Table 5.** Overview of data sources and sequencing.

Data sources and measures	Number of items	Source and scoring	Timing
Participant survey data			
Connection to services	46	CLOE^a^ program of research; all items developed for CLOE research study drawing on previous research and advisory committee consultation [14] These items are self-reported dichotomous questions (yes or no) concerning whether or not the participant has a current care provider or support worker, feeling confident, comfortable attending to care, feeling respected, and listened to. Other items are numerical answers concerning the frequency of use of primary care, emergency health care, social, and legal services in the prior 3 months	T0, T1, T2, T3
Safety	6	CLOE program of research; all items developed for CLOE research study based on previous research [14] and in consultation with the community advisory committee Items are self-reported sense of safety 1‐5, with 1 being not feeling safe at all and 5 feeling extremely safe. Safety level is assessed in home, building, neighborhood, relationships, and with health and social service encounters	T0, T1, T2, T3
Overall trust in outreach program	1	CLOE program of research; 5-point Likert with 1 no trust to 5 extreme trust. Developed for the CLOE research study as a result of earlier pilot work [54]	T1, T2, T3
Trust “scale”	8	CLOE Program of Research; developed for testing in the current study based on review of literature and previous research by team members Trust will be measured using a pilot scale developed from prior team research and literature; comprising 8 items rated on a 5-point Likert scale, anchored at 1 (*no trust*) and 5 (*complete trust*). As this scale is newly developed and has not yet undergone full psychometric validation, definitive cut-off scores have not been established; further testing is required to determine thresholds for clinical or practical interpretation [54]	T1, T2, T3
Demographics	33	CCHS^b^ Annual Component 2023 [60] Financial Strain Index [61] Other items developed for CLOE research study pertain directly to financial and food security, using 4-point Likert scales for each item where 1 is zero strain and 4 is extreme strain. Other financial items include average monthly household income and dichotomous item for receipt of government financial aid	T0, T1, T2, T3
Health	23	Patient Health Questionnaire for Anxiety and Depression [62] CCHS-General Health, Health Utility Index; and activities of daily living [60] Other items developed for CLOE research study pertain to the use of unregulated drugs and receipt of substitution therapies. All are dichotomous (yes or no). These items were developed in partnership with community advisors	T0, T1, T2, T3
Family and social network	9	CCHS Social Provisions Scale (CCHS-SPS) [60] UCLA^c^ Loneliness Scale (Version 3) [63]	T0, T1, T2, T3
Housing	35	Housing Instability Index [64] Housing Security Scale [65] Other items developed for CLOE research study based on unique context of women’s housing security reported in other research but not regularly included in existing measures. Such items attend to the quality of housing. These include dichotomous items (yes or no) on self-reported privacy in home, ability to prepare a meal, store food, and control who enters the home	T0, T1, T2, T3
Case notes			
Participant engagement notes	50	CLOE program of research; all items developed for CLOE research study (Multimedia Appendix 1: Participant engagement notes)	Ongoing, throughout intervention
Service provider notes	14	CLOE program of research; all items developed for the CLOE research study (Multimedia Appendix 1: Service provider notes)	Ongoing, throughout intervention
Interviews			
Contextual factors influencing engagement with CLOE intervention and health and social services	9	Interview guides developed for the CLOE research study drawing on expertise of team and review of literature (Multimedia Appendix 2)	T3

a CLOE: community-led outreach and engagement.

b CCHS: Canadian Community Health Survey.

c UCLA: University of California, Los Angeles.

#### Participant Case Notes

Outreach workers will document participants’ engagement in the intervention using Research Electronic Data Capture (REDCap) tools, hosted at the Capacity Research Centre at the University of British Columbia [66,67]. Participant case notes include three types of information: (a) records of each one-to-one engagement with a participant referred to as participant engagement notes; (b) quarterly reassessment of goals and progress; and (c) service provider notes that record IOW engagements with other service providers, either with or on behalf of a participant. The case note data entry matrix was developed drawing on previous research and includes structured (ie, quantitative) and unstructured open response elements (ie, qualitative) (Multimedia Appendix 1). Participant engagement notes include time, date, and mode of engagement (eg, in-person, via phone). IOWs will also record activities undertaken within 1 of the 10 core domains of outreach support detailing the purpose of the engagement, activities undertaken, and follow-up plans as appropriate. The quarterly reassessment and check-ins include open responses detailing outcomes of collaborative review of previously identified goals, the types of activities engaged in thus far, and outcomes of discussions between IOWs and participants concerning any noted revision to goals and/or plans for future engagement activities. Service provider notes follow a similar pattern to participant engagement notes, with explicit details recorded about the type of provider engaged with, the rationale, outcomes, and follow-up as appropriate.

#### Surveys

Baseline surveys (T0) will be facilitated by a trained research staff member and will occur within 1 week of participant enrollment. Survey variables include: demographic information; housing security; self-reported safety at home and community; food security; financial security; mobility; mental and physical health; family or social network; health and social care access; and sociolegal care. As noted in Table 5, some items have been specifically developed for the purpose of this study and others from existing measures.

Participants will be invited to participate in follow-up surveys facilitated by a trained research staff member at 4 months (T1), 8 months (T2), and 12 months (T3) postenrollment. The follow-up survey contains the same questions as the baseline survey and additional items to assess overall trust in the program, dimensions of trust, and participants’ access to supports over time. Table 5 provides an overview of survey items, case notes, and interviews, including item and question sources. Brief details are also provided for scoring.

Research staff will capture participants’ survey responses on REDCap [66,67], or on paper-based survey instruments. Data collected on the paper-based instrument will be entered into SPSS (IBM Corp.) and later compiled with the data to be exported from the REDCap database.

#### Qualitative Interviews

Enrolled participants will be invited to participate in one 1‐1 semistructured interview with a research staff member during the last month of the intervention period (T3). Interview guides have been developed based on research team expertise and previous research concerned with barriers and facilitators to women’s engagement with health and social services inclusive of the need for strengths-based, women-led approaches to care provision (Multimedia Appendix 2). Questions will attend to individuals’ choices concerning engagement, their perspectives about the quality and value of the relationship with the IOWs, and their experiences of structural factors, such as poverty, service accessibility, competing health concerns, and food and financial insecurity known to impact care engagement. Interviews will be recorded and audio recordings will be transcribed for data coding and analysis.

### Analysis

In accordance with convergent mixed methods research, quantitative and qualitative data sources will initially be analyzed independently (ie, in parallel) prior to data integration [55,56].

#### Quantitative Data

Survey and quantitative case note data will be managed in SPSS, and analysis will be primarily descriptive and exploratory recognizing the limited sample size, absence of a control group, and the aim of the project to examine trends over time. Specifically, descriptive statistics such as frequencies, means, and SDs will be used to summarize participant demographic characteristics, self-reported health status, and access to health and social services. Descriptive statistics, spaghetti plots, and boxplots [68,69] will be used to analyze trends over time in trust, safety, and access to health and social care services. Frequencies will also be calculated to describe the number and focus of participant engagements with outreach workers and provider engagements.

#### Qualitative Data

Interview and open text data will be managed in NVivo (QSR International) and analyzed using reflexive thematic analysis (RTA) methods [70,71] In keeping with RTA, the researchers’—inclusive of research partners’—roles in knowledge coproduction and the interpretive nature of coding are recognized. Interpretive coding necessitates integration of the theoretical perspectives into the analytic process, which in this study includes critical feminist perspectives concerning marginalization and GBV (see the Theoretical Approaches section). Thus, both inductive and deductive approaches to coding will be used to allow us to examine how interpersonal and structural (ie, marginalizing) circumstances influence IOW-participant relationships, including how outreach services are organized and implemented and how poverty and historical and ongoing GBV intersect to shape relationships over time. We will also attend to the contextual features that impact participants’ safety and engagement with health and social services to address their priority concerns. Study team members will read and reread interviews focusing on context and experiences. Meetings will be held to review and discuss the data to generate themes. Themes within RTA are the analytic outputs that represent patterns unified by a shared concept within and across the interview and case note data. As analysis progresses, we will identify analytic themes that provide a rich and nuanced understanding of how participants experience the intervention and factors impacting trust, safety planning, and care engagement. In keeping with RTA aims to provide rich and nuanced understanding of patterns and variations within a specific context, analysis will be further shaped by our commitment to data adequacy. Data adequacy approaches to analysis are appropriate when the aim is not to exhaustively enumerate descriptive themes (ie, saturation) and when more interpretive approaches are used in health services research [70,71]. As our aims are to interrogate how marginalization, context, and structural conditions shape participants’ experiences of trust, access, and safety, adequacy will be determined by analytic depth, reflexive rigor, and the extent to which the analysis produces actionable insights for health services design and delivery [70-72].

#### Data Integration

Merging, defined as bringing multiple databases together to be compared and interpreted together, will be the primary method used for data integration [55]. Merging occurs once the initial quantitative and qualitative analysis has occurred and most often includes a thematic approach to data integration and reporting of the findings that are explicitly in accordance with the study objectives [55]. Thus, the data will be integrated to construct themes related to the study core objectives concerning trends in trust, safety planning, and access to health and social services [55]. An additional thematic category will be created to describe the study participants’ demographic characteristics to nuance our understanding of participants’ social groupings across categories of age, ability, ethnicity, financial and housing security, and historical experiences accessing care. This demographic theme will allow greater understanding of similarities, differences, and severity of the marginalization experienced by participants. Using a thematic approach, we will identify key trends observed over time and the contextual factors that help nuance these trends in trust, safety, and care access. Specifically, survey findings across time points will be compared with interview and case note analyses concerning participants’ experiences in the program and contextual factors that help to provide greater understanding of how and potentially why such trends were observed. We will pay particular attention to similarities and differences (if observed) between the various findings across the diverse data sets to support a richer understanding of the findings observed. While the full nature of analysis will be dependent on observed trends over time, these thematic categories will provide an analytic framework to examine study outcomes in trends over time that are nuanced to the structural and interpersonal features of the participants’ experiences, their choices in program engagement, and structural features of the participants’ everyday lives that shape their engagement with the outreach workers, trust, safety, and access and receipt of health and social care. Visual representations of the themes concerning trust, safety planning, and health and social care access and receipt will be used to illustrate the study findings and integration results.

### Ethical Considerations

The study protocol was approved by the Behavioral Ethics Review Boards at the University of British Columbia (H23-02810; H23-03111; H24-01477), the University of Windsor (23‐202; 23‐203; 24-177), and Dalhousie University (2023‐6999; 2023‐7000). Prior to data collection, all participants will provide written informed consent to participate in the research. Financial honoraria will be provided to all participants for surveys ($30 CAD [US $21.53]) and qualitative interviews ($50 CAD [US $35.88]). Only researchers involved directly with this study will have access to encoded data. Findings will be presented to stakeholders through community reports, policy briefs, and at national and international conferences, as well as published in peer-reviewed scientific journals.

## Results

The study was funded by the Social Sciences and Humanities Research Council and Michael Smith Health Research BC in April 2019. Following ethics committee approval and extensive consultation with lead partner organizations and community engagement, intervention implementation began in October 2023 at study Hub A and in October 2024 at study Hub B. Participant enrollment began in November 2023 and closed in June 2025, and a total of 86 women were enrolled during that time. Data collection is ongoing and is expected to be finalized in January 2026. Data analysis will begin after January 2026, and results are expected to be submitted for publication and shared in community forums beginning in late spring 2026.

## Discussion

### Study Contributions

This study examines the implementation of a strengths-based, trauma- and violence-informed outreach intervention for women experiencing GBV who are not well-connected to health and social services. We anticipate that relational outreach, grounded in harm reduction and respect for self-determination, will support the development of trust between participants and outreach teams and may be associated with increased engagement with health and social services and strengthened safety planning over time. The mixed methods design is expected to generate insight into contextual and structural factors shaping these trends, contributing evidence that extends beyond behavioral explanations of care engagement.

Existing research suggests that outreach-based service delivery models can improve engagement among populations experiencing structural marginalization, including individuals affected by homelessness, mental health challenges, and substance use-related harms [16-20]. Outreach interventions addressing IPV have similarly reported improvements in psychosocial functioning and safety-related outcomes [24,28,29]. However, much of this literature conceptualizes engagement primarily through behavioral or individual-level determinants and gives limited attention to the structural conditions shaping service access and use. As a result, the roles of intersecting inequities—such as racism, sexism, poverty, and disability—in shaping engagement processes remain underexamined.

This study extends prior work by embedding outreach within a trauma- and violence-informed care framework and explicitly situating engagement within interpersonal and structural contexts. In contrast to approaches focused narrowly on IPV or postseparation support, the protocol encompasses diverse manifestations of GBV among women experiencing intersecting social and economic disadvantages. By integrating quantitative indicators of engagement with qualitative exploration of lived experiences, the study seeks to advance conceptualization of outreach beyond an individual-level intervention toward understanding it as a relational and structurally mediated health service strategy.

Furthermore, the study incorporates outcome domains that are less frequently operationalized in outreach research, including trust in providers, navigation of fragmented service systems, and safety planning processes. Examining these dimensions alongside contextual accounts may generate insight into mechanisms through which outreach contributes to engagement and well-being. This multidimensional approach positions the research to contribute to methodological and conceptual gaps in the literature by foregrounding the relational dynamics, system-level barriers, and structural determinants that shape outreach effectiveness, thereby informing the design of more equitable service delivery models.

### Strengths and Limitations

The study’s convergent mixed methods design enables integration of quantitative survey data with qualitative interviews and case-note documentation, supporting a nuanced examination of observable trends, participant experiences, and factors impacting trends [55]. Multisite implementation enhances contextual depth and allows exploration of variation across service settings [56]. Grounding the intervention in trauma- and violence-informed and harm reduction principles aligns with current best practices for engagement with populations affected by GBV [41-43].

As an exploratory protocol without a comparison group, the study will not support causal inference regarding intervention effectiveness, which, as noted earlier, is beyond the scope of an exploratory study. Sample size and potential attrition may limit statistical power and longitudinal interpretation. Findings may not be generalizable beyond similar programmatic or geographic contexts. Additionally, reliance on self-reported and program-generated data introduces the possibility of recall and reporting bias [56]. However, the inclusion of multiple data sources and time points can help alleviate potential bias.

### Future Directions

Findings from this study will inform subsequent research examining outreach interventions for women experiencing GBV. Future work may include larger-scale or controlled evaluations to assess effectiveness and implementation outcomes, as well as adaptations across additional settings or populations facing intersecting structural inequities. Further development of measurement approaches capturing trust, relational engagement, and structural determinants of care access may also be warranted. Beyond research implications, study insights may inform program and policy development by supporting integrated, nonsiloed service delivery models that prioritize relational engagement and trauma- and violence-informed principles [14].

### Knowledge Mobilization Plan

Study findings will be disseminated through academic, practice, and community-focused channels. Academic dissemination will include peer-reviewed publications and conference presentations. Practice-oriented outputs will be developed for service providers and community organizations, including brief reports, presentations, or webinars to support knowledge translation into program contexts. Community-engaged dissemination strategies will include plain-language summaries and stakeholder discussions to enhance accessibility and relevance for participating organizations and communities. Policy-relevant outputs may also be prepared to inform decision-makers regarding service integration and outreach approaches addressing GBV and structural barriers to care.

## Supplementary material

10.2196/88265 Multimedia Appendix 1 Case note example.

10.2196/88265 Multimedia Appendix 2 Interview guide example.

## Abbreviations

CLOE: community-led outreach and engagement
GBV: gender-based violence
IOW: intervention outreach worker
IPV: intimate partner violence
REDCap: Research Electronic Data Capture
RTA: reflexive thematic analysis
