# Supplementary material for: Trends in Trust, Safety, and Health Service Access Among Women Participating in an Antiviolence Outreach Program: Protocol for a Mixed Methods Study
Source: JMIR Res Protoc. 2026 Apr 27;15:e88265. doi: 10.2196/88265 (PMC13119384; doi:10.2196/88265)
Supplement: Multimedia Appendix 1 [file resprot-v15-e88265-s001.pdf]

THE CLOE PROJECT

# Appendix 1: Case Note Examples

## Participant Engagement Notes

| | | |
|---|---|---|
| Record ID | ______________________ | |
| **Overview** | | |
| Date | ______________________ | Required |
| Worker Name | ❍ Outreach Worker 1<br>❍ Outreach Worker 2 | Required |
| Mode of Contact | ❍ Phone Call<br>❍ In Person<br>❍ Text<br>❍ Email | |
| Connection Made | ❍ Yes<br>❍ No | |
| Missed Connection<br><br>Select N/A when there was NO plan to meet/connect | ❍ Yes<br>    ❍ By Outreach Worker<br>    ❍ By Participant<br>❍ N/A | Only asked if Connection Made = No |
| Interaction Focus<br><br>What was the focus of this interaction? (please check all that apply) | ❑ Scheduling/Confirmation of Appointment with Client<br>❑ Priorities<br>❑ Emotional Support<br>❑ Crisis Management<br>❑ Service Declined | Only asked if Connection Made = Yes |
| Scheduling Notes | ______________________ | Only asked if Interaction Focus = Scheduling/ Confirmation of Appointment with Client |
| Notes if Client Declined Interaction/Service | ______________________ | Only asked if Interaction Focus = Service Declined |

## Crisis Management

| | | |
|---|---|---|
| **Nature of Crisis**<br>What is the nature of the crisis? Please provide a BRIEF description. | ______________________ | Only asked if Interaction Focus = Crisis Management |
| **Crisis Intervention Provided**<br>In response to their crisis, which organization(s) are you connecting with on this participant's behalf? | ______________________ | Only asked if Interaction Focus = Crisis Management |

## Priorities

| | Explored or Worked on | |
|---|---|---|
| Financial Security | ❍ | |
| Education/Vocational | ❍ | |
| Food Security | ❍ | |
| Housing | ❍ | |
| Transportation Security | ❍ | Only asked if Interaction Focus = Priorities |
| Legal | ❍ | |
| Family/Social Network | ❍ | |
| Substance Use | ❍ | |
| Health | ❍ | |
| Safety | ❍ | |

## Financial Security

| | | |
|---|---|---|
| Financial Security Goals | ❑ Apply for social assistance<br>❑ Move from one type of assistance to another (e.g., Ontario Works (OW) to ODSP)<br>❑ Tax preparation<br>❑ Banking support<br>❑ Find employment/peer work<br>❑ Other | Only asked if Priorities, Financial Security = Explored or Worked on |
| Financial Security Activities | ❑ Provided Education or Information<br>❑ Made Referral(s) or Applied<br>❑ Accompaniment<br>❑ Planned an Accompaniment<br>❑ Safety Planning<br>❑ Other | Only asked if Priorities, Financial Security = Explored or Worked on |

| | | |
|---|---|---|
| Next Steps Related to Financial Security | ________________ | Only asked if Priorities, Financial Security = Explored or Worked on |
| **Education/Vocational** | | |
| Education or Vocational Goals | ❑ Find/apply for educational funds<br>❑ Find educational or training programs<br>❑ Other | Only asked if Priorities, Education/Vocational = Explored or Worked on |
| Education or Vocational Activities | ❑ Provided Education or Information<br>❑ Made Referral(s) or Applied<br>❑ Accompaniment<br>❑ Planned an Accompaniment<br>❑ Safety Planning<br>❑ Other | Only asked if Priorities, Education/Vocational = Explored or Worked on |
| Next Steps Related to Education/Vocational | ________________ | Only asked if Priorities, Education/Vocational = Explored or Worked on |
| **Food Security** | | |
| Food Security Goals | ❑ Get information about food bank, meal program, etc.<br>❑ Referral or sign up with meal/grocery program<br>❑ Other | Only asked if Priorities, Food Security = Explored or Worked on |
| Food Security Activities | ❑ Provided Education or Information<br>❑ Made Referral(s) or Applied<br>❑ Accompaniment<br>❑ Planned an Accompaniment<br>❑ Safety Planning<br>❑ Other | Only asked if Priorities, Food Security = Explored or Worked on |
| Next Steps Related to Food Security | ________________ | Only asked if Priorities, Food Security = Explored or Worked on |

## Housing

| | | |
|---|---|---|
| Housing Goals | ❑ Change current situation<br>❑ Leave the neighbourhood<br>❑ Need emergency housing<br>❑ Landlord relationship & communication<br>❑ Acquire household necessities<br>❑ Other | Only asked if Priorities, Housing = Explored or Worked on |
| Details Related to Change Current Situation<br><br>Please describe what needs to be changed about participant's current situation. This could be about a change to their current housing or a move or transfer. | | Only asked if Housing Goals = Change Current Situation |
| Housing Activities | ❑ Provided Education or Information<br>❑ Made Referral(s) or Applied<br>❑ Accompaniment<br>❑ Planned an Accompaniment<br>❑ Safety Planning<br>❑ Sanitation, Pest Control or Maintenance<br>❑ Other | Only asked if Priorities, Housing = Explored or Worked on |
| Next Steps Related to Housing | | Only asked if Priorities, Housing = Explored or Worked on |

## Transportation Security

| | | |
|---|---|---|
| Transportation Security Goals | ❑ Secure immediate transportation (bus ticket or taxi voucher)<br>❑ Secure ongoing transportation (bus pass, subsidy, etc.)<br>❑ Increase confidence and independence in transportation options<br>❑ Other | Only asked if Priorities, Transportation Security = Explored or Worked on |
| Transportation Security Activities | ❑ Provided Education or Information<br>❑ Made Referral(s) or Applied<br>❑ Accompaniment<br>❑ Planned an Accompaniment<br>❑ Safety Planning<br>❑ Other | Only asked if Priorities, Transportation Security = Explored or Worked on |

| | | |
|---|---|---|
| Next Steps Related to Transportation Security | | Only asked if Priorities, Transportation Security = Explored or Worked on |
| **Legal** | | |
| Type of Legal Services/Supports | ❑ Family Legal Services<br>❑ Criminal Legal Services<br>❑ Other Legal Services | Only asked if Priorities, Legal = Explored or Worked on |
| Family Legal – Goals | ❑ Find a legal service or lawyer<br>❑ Change current lawyer or legal service<br>❑ Find legal education<br>❑ Prepare for appearance (in court or legal office)<br>❑ Access notary or other administrative support<br>❑ Other | Only asked if Family Legal = Checked |
| Criminal Legal – Goals | ❑ Find a legal service or lawyer<br>❑ Change current lawyer or legal service<br>❑ Find legal education<br>❑ Prepare for appearance (in court or legal office)<br>❑ Access notary or other administrative support<br>❑ Other | Only asked if Criminal Legal = Checked |
| Other Legal – Goals | ❑ Identification<br>❑ Find a legal service or lawyer<br>❑ Change current lawyer or legal service<br>❑ Find legal education<br>❑ Prepare for appearance (in court or legal office)<br>❑ Access notary or other administrative support<br>❑ Other | Only asked if Other Legal = Checked |
| Identification Type | ❑ ON ID or Driver's License<br>❑ ON Health Card<br>❑ Birth Certificate<br>❑ Other | Only asked if Other Legal Goals = Identification |
| Other Type of ID | | Only asked if Identification Type = Other |

| | | |
|---|---|---|
| Legal Activities | ❑ Provided Education or Information<br>❑ Made Referral(s) or Applied<br>❑ Accompaniment<br>❑ Planned an Accompaniment<br>❑ Safety Planning<br>❑ Filed a protection order<br>❑ Other | Only asked if Priorities, Legal = Explored or Worked on |
| Next Steps Related to Legal Issues | | Only asked if Priorities, Legal = Explored or Worked on |
| **Family/Social Network** | | |
| Family/Social Network Goals<br><br>Include any goals related to engaging or re-engaging with children, other family members or social network, preventing contact, etc. | | Only asked if Priorities, Family/Social Network = Explored or Worked on |
| Family/Social Network Activities | ❑ Provided Education or Information<br>❑ Made Referral(s) or Applied<br>❑ Accompaniment<br>❑ Planned an Accompaniment<br>❑ Safety Planning<br>❑ Other | Only asked if Priorities, Family/Social Network = Explored or Worked on |
| Next Steps Related to Family/Social Network | | Only asked if Priorities, Family/Social Network = Explored or Worked on |
| **Substance Use** | | |
| Substance Use Goals | ❑ Reduce or change use<br>❑ Harm reduction & OD prevention<br>❑ Detox<br>❑ Treatment<br>❑ Support program (i.e., self-help group or counselling)<br>❑ Maintenance or safe supply program<br>❑ Other | Only asked if Priorities, Substance Use = Explored or Worked on |

| | | |
|---|---|---|
| Substance Use Activities | ❑ Provided Education or Information<br>❑ Made Referral(s) or Applied<br>❑ Accompaniment<br>❑ Planned an Accompaniment<br>❑ Safety Planning<br>❑ Other | Only asked if Priorities, Substance Use = Explored or Worked on |
| Next Steps Related to Substance Use | ______________________ | Only asked if Priorities, Substance Use = Explored or Worked on |
| **Health** | | |
| Health Goals/Needs | ❑ Physical Health<br>❑ Mental Health | Only asked if Priorities, Health = Explored or Worked on |
| Physical Health Goals | ❑ Find or change primary care provider<br>❑ Find or change a specialist provider<br>❑ Assistance arranging appointments<br>❑ Assistance getting to appointments<br>❑ Other | Only asked if Health Goals = Physical Health |
| Mental Health Goals | ❑ Find or change mental health provider<br>❑ Assistance arranging appointments<br>❑ Assistance getting to appointments<br>❑ Other | Only asked if Health Goals = Mental Health |
| Health Activities | ❑ Provided Education or Information<br>❑ Made Referral(s) or Applied<br>❑ Accompaniment<br>❑ Planned an Accompaniment<br>❑ Safety Planning<br>❑ Other | Only asked if Priorities, Health = Explored or Worked on |
| Next Steps Related to Health | ______________________ | Only asked if Priorities, Health = Explored or Worked on |

## Safety

| | | |
|---|---|---|
| Goals for Safety | ❑ Prevent and address intimate partner violence<br>❑ Feel safe in the community<br>❑ Feel safe at home<br>❑ Feel safe when accessing health and social services<br>❑ Other | Only asked if Priorities, Safety = Explored or Worked on |
| Other Safety Goals<br><br>If other, what else is important to you? | ______ | Only asked if Goals for Safety = Other |
| Activities Related to Safety | ❑ Provided Education or Information<br>❑ Made Referral(s) or Applied<br>❑ Accompaniment<br>❑ Planned an Accompaniment<br>❑ Safety Planning<br>❑ Other | Only asked if Priorities, Safety = Explored or Worked on |
| Next Steps Related to Safety | ______ | Only asked if Priorities, Safety = Explored or Worked on |

## End of Interaction

| | | |
|---|---|---|
| Total Time Spent (in minutes) | ______ | Only asked if Connection Made = Yes |
| Overall Notes | ______ | Not asked if Interaction Focus is only Scheduling/ Confirmation of Appointment with Client |
| Next Steps/Follow-up Plan | ______ | Not asked if Interaction Focus is only Scheduling/ Confirmation of Appointment with Client |

## Service Provider Notes

| | | |
|---|---|---|
| Record ID | ___________________________ | |
| Contact Date | ___________________________ | Required |
| Worker Name | ❍ Outreach Worker 1<br>❍ Outreach Worker 2 | Required |
| Name of Person Contacted | ___________________________ | |
| Role of Person Contacted | ___________________________ | |
| Organization Name | ❍ [hosting community organization]<br>❍ Other: ________________________ | |
| Organization Type | ___________________________ | Only asked if Organization Name = Other |
| Mode of Contact | ❍ Phone Call<br>❍ Face-to-Face<br>❍ Text<br>❍ Email<br>❍ Other: _______________ | |
| Contact Made | ❍ Yes<br>❍ No | |
| Participant Involvement | ❍ Participant Participated<br>❍ On Behalf of Participant | |
| Reason for Engagement | ___________________________ | |

| | | |
|---|---|---|
| Engagement Goal Achieved | ❍ Yes<br>❍ No | Only asked if Contact Made = Yes |
| Summary of Engagement | ________________ | |
| Next steps/follow-up plan | ________________ | |
