# Supplementary material for: Trends in Trust, Safety, and Health Service Access Among Women Participating in an Antiviolence Outreach Program: Protocol for a Mixed Methods Study
Source: JMIR Res Protoc. 2026 Apr 27;15:e88265. doi: 10.2196/88265 (PMC13119384; doi:10.2196/88265)
Supplement: Multimedia Appendix 2 [file resprot-v15-e88265-s002.pdf]

Appendix 2: Interview Guide Example

Interview Guide: End of Project

Date:
Interviewer:
Participant ID:

Thank you for taking the time to meet with me and agreeing to do this interview. We are hoping to hear more from you about your experience engaging with the CLOE Project. We want to learn about what you think worked well and any areas of improvement that could be important for you and other women.

I won't be asking questions that evaluate your outreach worker or their performance, and nothing you say today will be shared back directly with them. Just like with your survey, anything you say here will be kept confidential.

---

1. Learning about what women were hoping to gain from the program is helpful for future programs like CLOE. **Thinking about when you first heard about CLOE, can you tell me more about what you were hoping to get out of the project?** It might be useful to think about why you agreed to participate or a bit about what was going on in your life at the time that made you think, "hey, this might be useful for me."
2. One of the things we are also trying to learn about is what the project looked like 'on the ground' for women. There is no right or wrong answer to this question. **Could you tell me about how you and your outreach worker worked together? What did that process look like for you?**
3. We are also interested in learning about **what worked well** for people participating in the project. **Please tell me a little bit about what being in this project has meant to you. How helpful has the project been for you? What's been the best part or what benefits have you seen?**
4. **Were there any challenging parts or things that made it hard for you to participate?**
5. This project layered outreach services on to what was already offered at [name of organization]. **What (if anything) do you think that CLOE added, beyond what was already being done in your local community?**
6. If we were starting over, **what do you think we could add to CLOE or should do differently?**
7. **We would like to know about how the end of your participation with CLOE is impacting you.** We know that often women don't get a chance to talk about the end or a loss of a service like this. **Could you please tell me your thoughts on this ending?**
8. To wrap up, **what other thoughts or ideas do you have about this project?** For example, is there anything else you think we should know about how to better support women to engage with services in the local area or community?

THANK YOU
